# IS THERE A RELATIONSHIP BETWEEN INTESTINAL FRUCTOSE MALABSORPTION, GASTROINTESTINAL SYMPTOMS AFTER FRUCTOSE OVERLOAD, AND BODY ADIPOSITY IN ADOLESCENTS?

**DOI:** 10.1590/S0004-2803.24612025-078

**Published:** 2026-05-22

**Authors:** Dayane Pêdra Batista de FARIA, Ana Paula Bidutte CORTEZ, Ricardo Palmero OLIVEIRA, Maria Sylvia de Souza VITALLE, Patrícia da Graça Leite SPERIDIÃO, Mauro Batista de MORAIS

**Affiliations:** 1 Universidade Federal de São Paulo, Gastroenterologia Pediátrica, São Paulo, SP, Brasil.; 2 Universidade Federal de São Paulo, Escola Paulista de Medicina, Programa de Pós-Graduação em Nutrição, São Paulo, SP, Brasil.; 3 Universidade Federal de São Paulo, Escola Paulista de Medicina, Centro de Atendimento e Apoio ao Adolescente (CAAA), São Paulo, SP, Brasil.

**Keywords:** Fructose malabsorption, fructose intolerance, adolescents, bioelectrical impedance analysis, intestinal permeability, adiposity, hydrogen breath test, Má absorção intestinal de frutose, intolerância à frutose, adolescentes, análise da impedância bioelétrica, permeabilidade intestinal, adiposidade, teste respiratório do hidrogênio no ar expirado

## Abstract

**Background:**

Over the past several decades, dietary fructose intake has increased significantly, along with the prevalence of obesity. Fructose malabsorption is associated with gastrointestinal symptoms and may lead to fructose intolerance. Evidence suggests a probable association between intestinal fructose malabsorption and increased body adiposity.

**Objective:**

To analyze the prevalence of intestinal fructose malabsorption and intolerance in asymptomatic adolescents and to investigate their relationship with fructose intake, body adiposity, systemic metabolic indicators, and intestinal permeability.

**Methods:**

This cross-sectional observational study included 37 adolescents without gastrointestinal symptoms. Fructose malabsorption was investigated using the hydrogen breath test along with the lactulose breath test. Food intake was evaluated using a habitual food survey. Nutritional status was assessed by measuring weight, height, skin folds, abdominal circumference, and via Bioelectrical Impedance Analysis (BIA). Systemic metabolic indicators and serum zonulin levels were also evaluated to determine intestinal permeability.

**Results:**

Of the 37 adolescents tested 16 (43.2%) exhibited fructose malabsorption. Gastrointestinal symptoms compatible with fructose intolerance were reported by 8 (50.0%) of the 16 adolescents with fructose malabsorption, and by 5 (23.8%) of the 21 adolescents without fructose malabsorption (*P*=0.095). Fructose intake among adolescents with and without fructose malabsorption was 7.5 and 7.9 g/day, respectively (*P*=0.807). Body adiposity was comparable across groups in both females (34.2% and 33.2%; *P*=0.738) and males (21.0% and 22.2%; *P*=0.731). Metabolic indicators and zonulin levels were similar, except for urea, which was significantly higher in the malabsorption group (31.7±6.3 mg/dL) compared to those without fructose malabsorption (24.5±÷7.8 md/dL; *P*=0.012).

**Conclusion:**

Intestinal fructose malabsorption is common in asymptomatic adolescents and is not associated with adiposity, biochemical changes, or increased intestinal permeability. However, it correlates with elevated fermentation in the lactulose breath test. Fructose intolerance is linked to presence gastrointestinal symptoms following lactulose ingestion.

## HIGHLIGHTS

• Intestinal fructose malabsorption is common in asymptomatic adolescents and it is not always associated with fructose intolerance

• Intestinal fructose malabsorption is not associated with adiposity, biochemical changes, or increased intestinal permeability.

• The area under the curve showed that adolescents with fructose malabsorption produced more hydrogen during the lactulose breath test

• There is concordance between fructose intolerance and gastrointestinal symptoms following lactulose ingestion.

## RESUMO

**Contexto:**

Nas últimas décadas, a ingestão de frutose na dieta aumentou significativamente, juntamente com a prevalência da obesidade. Má absorção de frutose está associada a sintomas gastrointestinais e pode levar à intolerância à frutose. Evidências sugerem uma provável associação entre a má absorção intestinal de frutose e o aumento da adiposidade corporal.

**Objetivo:**

Analisar a prevalência de má absorção intestinal e intolerância à frutose em adolescentes assintomáticos e investigar sua relação com a ingestão de frutose, adiposidade corporal, indicadores metabólicos sistêmicos e permeabilidade intestinal.

**Métodos:**

Este estudo observacional transversal incluiu 37 adolescentes sem sintomas gastrointestinais. Má absorção de frutose foi investigada utilizando o teste respiratório de hidrogênio. Foi realizado, também, teste respiratório com lactulose. A ingestão alimentar foi avaliada por meio de um questionário sobre os hábitos alimentares. O estado nutricional foi avaliado por meio da mensuração de peso, altura, pregas cutâneas, circunferência abdominal e Análise de Impedância Bioelétrica (BIA). Foram avaliados, também, indicadores metabólicos sistêmicos e níveis séricos de zonulina como iondicador da permeabilidade intestinal.

**Resultados:**

Dos 37 adolescentes estudados, 16 (43,2%) apresentaram má absorção de frutose. Sintomas gastrointestinais compatíveis com intolerância à frutose foram relatados por 8 (50,0%) dos 16 adolescentes com má absorção de frutose e por 5 (23,8%) dos 21 adolescentes sem má absorção de frutose (*P*=0,095). A ingestão de frutose entre os adolescentes com e sem má absorção de frutose foi de 7,5 e 7,9 g/dia, respectivamente (*P*=0,807). A adiposidade corporal foi comparável entre os grupos, tanto em meninas (34,2% e 33,2%; *P*=0,738) quanto em meninos (21,0% e 22,2%; *P*=0,731). Os indicadores metabólicos e os níveis de zonulina foram semelhantes, com exceção da ureia, que foi significativamente maior no grupo com má absorção (31,7±6,3 mg/dL) em comparação com aqueles sem má absorção de frutose (24,5±7,8 mg/dL; *P*=0,012).

**Conclusão:**

A má absorção intestinal de frutose é comum em adolescentes assintomáticos e não está associada à adiposidade, alterações bioquímicas ou aumento da permeabilidade intestinal. No entanto, correlaciona-se com fermentação elevada no teste respiratório com lactulose. A intolerância à frutose está relacionada à presença de sintomas gastrointestinais após a ingestão de lactulose.

## INTRODUCTION

Fructose is one of the main monosaccharides in the diet. It can be fermented in the intestinal lumen when not absorbed. It therefore belongs to the group of FODMAPs: fermentable oligosaccharides, disaccharides, monosaccharides, and polyols^1^ ^-^ ^3^. The intestinal absorption capacity of fructose varies among the population^2^ ^,^ ^4^ ^,^ ^5^. Unabsorbed fructose, like other FODMAPs, can be associated with gastrointestinal symptoms^3^ ^,^ ^5^. When people consume more fructose than they can absorb, fructose intolerance can occur, characterized by abdominal distension, abdominal pain, flatulence, and diarrhea caused by the fermentation of unabsorbed fructose^2^ ^,^ ^3^ ^,^ ^6^ ^-^ ^10^.

In recent decades, there has been an increase in fructose consumption in several countries, coinciding with an increase in the prevalence of obesity^11^ ^,^ ^12^. The increase in fructose consumption is mainly due to industrialized products^13^ ^,^ ^14^. Fructose is present in natural foods such as fruits, vegetables, legumes, and honey, as well as in industrial additives, such as high-fructose corn syrup, created from the hydrolysis of sugar cane molasses. From the 1960s onwards, the food industry began to use fructose corn syrup in products such as soft drinks, sweets, and cakes due to its low cost, longer shelf life, and high sweetening power^2^ ^,^ ^3^.

In this context, obesity and other metabolic disorders such as type 2 diabetes mellitus, insulin resistance, and increased blood pressure have been attributed, at least in part, to increased fructose consumption^9^ ^,^ ^11^ ^,^ ^12^ ^,^ ^14^. Adolescents are included in this process, given that they have been consuming more fructose drinks in recent decades. The increase in this consumption occurred at the same time as the increase in the occurrence of excess weight^15^ ^,^ ^16^ high levels of serum triglycerides and retinol-binding protein 4^17^ as well as hyperinsulinemia^15^ ^,^ ^18^ ^.^ A study carried out in our department^10^ showed that children with intestinal fructose malabsorption had a high body mass index (BMI), suggesting the possibility of increased adiposity related to fructose malabsorption. This study also found that fructose malabsorption was associated with greater lactulose fermentation capacity in the breath test for hydrogen in exhaled air. This difference in fermentative capacity may be directly related to the characteristics of the intestinal microbiota, as demonstrated in American children with irritable bowel syndrome^9^. On the other hand, circulating levels of zonulin may be elevated in overweight children and adolescents, suggesting increased intestinal permeability associated with obesity^19^ ^-^ ^21^. To the best of our knowledge, no studies to date have evaluated serum zonulin levels in the context of intestinal fructose malabsorption.

Therefore, this study aimed to analyze the prevalence of intestinal malabsorption and fructose intolerance in asymptomatic adolescents, as well as to examine the relationship between fructose malabsorption, habitual fructose intake, body adiposity, and intestinal permeability.

## METHODS

### Study design

This is a quantitative, cross-sectional, and observational study with a non-probabilistic sampling (convenience sample) of 37 adolescents with no history of gastrointestinal symptoms. The study was carried out at the Adolescent Care and Support Center (CAAA) of the Federal University of São Paulo - Paulista School of Medicine. Upon admission, all the adolescents’ guardians and all the adolescents signed a Free and Informed Consent Form and a Free and Informed Assent Form, respectively. The study was approved by the Research Ethics Committee of the Federal University of São Paulo/EPM (CAEE: 83553818.6.0000.5505). The study was carried out between May 2019 and February 2020.

### Sample

All adolescents consecutively treated at the CAAA were invited to participate. Adolescents aged between 10 and 19 years without chronic or recurrent gastrointestinal symptoms were included. Adolescents with endocrinopathies, nephropathies, heart disease, illicit drug use, neurological involvement, pregnancy, energy-protein malnutrition, treatment with metformin, or medications that could influence short-term energy balance and with antibiotic use within the 30 days before enrollment were excluded.

### Data collection

All the adolescents completed a standardized questionnaire with demographic information (gender, age, economic status, and the clinical history regarding symptoms affecting the gastrointestinal tract or other organ systems). They were also asked about prior use of probiotics and antibiotics. A nutritional survey was performed based on the participants’ usual diet, and anthropometric measurements (weight, height, skinfolds, and circumferences). In addition, bioelectrical impedance and a hydrogen breath test with fructose and lactulose were carried out.

### Breath test for hydrogen in exhaled air with fructose and lactulose

Hydrogen breath tests with fructose and lactulose were carried out one week apart. Participants were advised to restrict the consumption of foods containing lactose, fructose, and dietary fiber on the day before each test. The test was carried out after fasting for 12 hours and cleaning the oral cavity with 0.05% chlorhexidine. Exhaled air samples were collected using a portable device (Easy H_2_® - DYNAMED, São Paulo, Brazil).

For the respiratory tests, an exhaled air sample was collected in a fasting state. Subsequently, 1 g of fructose/kg of body weight (maximum 50 g) was administered orally for the fructose breath test^10^ ^,^ ^22^ or 10 g of lactulose diluted in 150 mL of water for the lactulose breath test.

Exhaled air samples were collected 15, 30, 45, 60, 90, 120, 150, and 180 minutes after substrate ingestion. Any symptoms (abdominal distension, nausea, vomiting, excessive flatulence, and diarrhea) that might occur during sample collection were recorded. In addition, a follow-up call was made to check any symptom occurring between the end of the breath sample collection and 24 hours after ingestion of fructose or lactulose^7^ ^,^ ^10^ ^,^ ^23^.

Fructose malabsorption was characterized by an increase in hydrogen concentration ≥20 ppm compared to baseline in any of the samples collected until the end of the fructose breath test^10^ ^,^ ^22^. Fructose intolerance was characterized by the occurrence of gastrointestinal symptoms such as abdominal pain, flatulence, abdominal distension, diarrhea, nausea, and vomiting during the 24 hours following fructose ingestion^10^ ^,^ ^22^. Gastrointestinal symptoms after ingesting lactulose were also assessed using the same methodology as in the fructose test.

### Anthropometry, bioelectrical impedance analysis, and classification of nutritional status

Weight and height were measured using a digital scale (Filizola Balanças Industriais, São Paulo, Brazil) with a capacity of 150 kg and a sensitivity of 100 g. Height was measured using a fixed vertical stadiometer, measuring up to 190 cm and a sensitivity of 0.1 cm. The anthropometric indices used to classify nutritional status were the BMI-for-age (BMI/A) and height-for-age (H/A) z-scores, based on the z-score reference values proposed by the World Health Organization (2007)^24^. The AnthroPlus software version 1.0.3 (Geneva, Switzerland) was used for this analysis. Adolescents were classified as overweight (BMI/age z-score >+1 SD); and normal weight (BMI/age z-score between ≥ -2 SD and ≤+1 SD). The term “overweight” was used for adolescents who were overweight (>+1 SD and ≤+2 SD) or obese (>+2 SD).

The thickness (mm) of the skinfolds, including the subscapular and triceps, was measured using a Lange® adipometer. Measurements were taken according to the techniques traditionally recommended by Lohman et al. (1991)^25^. Arm, abdominal, and hip circumferences (cm) were measured using an inelastic tape measure, following the method described by Lohman et al. (1988)^26^. Three measurements were taken for each site, and the average was adopted as the final value. All measurements were performed by single evaluator.

To calculate body fat percentage, a Quantum 101 bioelectrical impedance device from RJL Systems Electrode Placement (RJL Systems, Miami, FL) was used, following the manufacturer’s guidelines. The fat percentage to characterize obesity was ≥30% for females and ≥25% for males^27^ ^,^ ^28^.

### Food intake

Dietary intake was assessed through habitual food surveys^29^ ^,^ ^30^, administered in a standardized format by an experienced nutritionist.

The interview detailed meal times, the foods or preparations consumed, and portion sizes, referring to the typical weekday diet. Macronutrient and micronutrient calculations were carried out using the computer program Avanutri® (Avanutri, Três Rios, Rio de Janeiro). The habitual intake of fructose was assessed using the Table of Chemical Composition of Foods (TABNUT), a web application reformulated and updated by the Department of Health Informatics of the Paulista School of Medicine/UNIFESP, which uses the reference standard adopted by the United States Department of Agriculture (USDA) as its database, version 25^31^.

### Metabolic indicators

Fasting for at least 8 hours was recommended for the collection of metabolic indicators. These tests were conducted at the São Paulo Central Hospital Laboratory according to routine analytical methods. Serum zonulin levels were measured by ELISA using the Elabscience kit (Texas, USA), following the manufacturer’s instructions.

### Statistical analysis

Continuous variables were presented as mean and standard deviation or as medians with 25th and 75th percentiles, depending on the distribution. Categorical variables were expressed in percentages. The tests used are indicated with the results, considering a significance level of 5%. Statistical analysis was performed using SigmaStat - Sigma Plot version 11.0 (Systat Software, San Jose, CA, USA).

## RESULTS

The study included 37 adolescents with no history of gastrointestinal symptoms. The respiratory test after fructose administration showed that 16 (43.2%) of the 37 adolescents had fructose malabsorption. The dose of fructose administered in the breath test was similar (*P*=0.501) in adolescents with (47.6±4.2 g) and without (48.6±4.2 g) fructose malabsorption. Gastrointestinal symptoms consistent with fructose intolerance were observed in 8 (50.0%) of the 16 adolescents with fructose malabsorption, and in 5 (23.8%) of the 21 adolescents without fructose malabsorption (*P*=0.095) during the 24 hours following fructose administration in the breath test. Among the adolescents with fructose malabsorption, 4 had abdominal pain, 2 reported abdominal pain and abdominal distension, 1 had abdominal pain and diarrhea, and 1 experienced nausea. Among those without fructose malabsorption, 2 experienced nausea, 1 had abdominal pain, distension, and diarrhea, 1 had abdominal pain and distension, and 1 had abdominal pain only. Table 1 shows the demographic characteristics and gastrointestinal symptoms according to the presence of intestinal fructose malabsorption.

**TABLE 1:** Age, gender, and gastrointestinal clinical manifestations in the fructose breath test according to the presence of intestinal fructose malabsorption.

Parameters	Fructose malabsorption		*P*
	Yes (16)	No (21)	
Age (years)	15.3±2.1	14.1±1.9	0.078^1^
Sex (male/female)	9/7	9/12	0.602^2^
Fructose breath test intolerance	8 (50.0%)	5 (23.8%)	0.095^1^
Clinical manifestation			
Abdominal pain	7	3	0.118
Abdominal distension	2	2	1.000^3^
Nausea	1	2	0.618^3^
Diarrhea	2	1	0.567^3^

¹Student’s t-test. ²Chi-square test. ^3^Fisher’s exact test.

In the respiratory test with lactulose, 5 adolescents were not hydrogen producers, while 32 produced hydrogen in the fructose test. Of the 5 adolescents who did not show hydrogen production in the lactulose test, 2 produced hydrogen in the fructose test.

In another 7 adolescents, a pattern compatible with bacterial overgrowth of the small intestine was observed during the respiratory test with lactulose indicated by an increase in hydrogen (>20 ppm) up to the sample collected 60 minutes after lactulose administration^10^ ^,^ ^22^. In the lactulose breath test, gastrointestinal symptoms (pain, distension, diarrhea, and nausea) were observed in 14 of the 37 adolescents. The Kappa coefficient showed substantial agreement (Kappa=0.65; 95% confidence interval: 0.39 to 0.90), i.e. 10 adolescents had symptoms in both tests, while 21 had no symptoms in either of the two respiratory tests.

**FIGURE 1:**
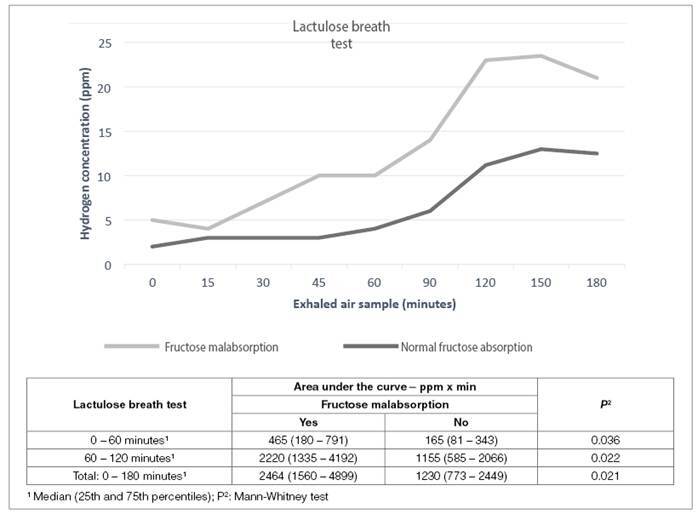
The median concentration of hydrogen in exhaled air and area under the curve after administration of lactulose during the respiratory test according to the presence of intestinal fructose malabsorption.

Figure 1 shows the median concentration of hydrogen in exhaled air from the lactulose breath test samples, according to the presence or absence of intestinal fructose malabsorption. The area under the curve showed that adolescents with fructose malabsorption produced more hydrogen during the first hour of the test (suggesting hydrogen production in the small intestine) and during the second and third hours (indicating hydrogen production in the colon), with a statistically significant difference (Figure 1). A correlation was also observed between the area under the hydrogen concentration curves in the fructose and lactulose tests (n=37; r = +0.48; *P*=0.003).

Table 2 shows the values for fructose and other macronutrient intake, anthropometric data, body mass index, and BIA. All results showed that the consumption of energy, proteins, lipids, and fructose was similar between the two groups. The same was observed for body adiposity parameters.

**TABLE 2:** Estimated energy, fructose and macronutrient intake, anthropometric data, and bioelectrical impedance according to the presence of intestinal fructose malabsorption.

Parameters	Fructose malabsorption		*P*
	Yes (16)	No (21)	
Food register			
Energy (kcal/day)	1511 (1375.1-1560.2)	1401.5 (1214.9-1863.3)	0.828^2^
Fructose (g)	7.5±5.4	7.9±4.8	0.807^1^
Fructose (g/kg/weight/day)	0.1 (0.0-0.2)	0.2 (0.0-0.2)	0.872^2^
Carbohydrates (g)	197.6±46.1	208.8±98.3	0.686^1^
Carbohydrates (g/kg/weight/day)	3.2 (2.3-4.5)	3.9 (2.3-5.1)	0.748^2^
Protein (g)	72.3±21.0	79.1±21.6	0.359^1^
Protein (g/kg/weight/day)	1.3±0.5	1.5±0.4	0.184^1^
Lipids (g)	42.0±13.1	43.9±24.9	0.784^1^
Lipids (g/kg/weight/day)	0.8±0.3	0.8±0.4	0.722^1^
Fiber (g)	15.3 (11.2-21.1)	15.1 (13.2-23.1)	0.630^2^
**Anthropometric data**			
Height (cm)	1.60 (1.54-1.66)	1.61 (1.54-1.71)	0.591^2^
Weight (kg)	59.60±17.7	60.37±18.24	0.898^1^
BMI (kg/m^2^)	22.95±6.12	22.86±5.86	0.986^1^
H/A Z scores	-0.49±1.2	0.26±1.11	0.055^1^
BMI/A Z scores	0.59±1.40	0.83±1.34	0.598^1^
Arm circumference (cm)	27.48±6.0	26.85±4.6	0.723^1^
Abdominal circumference (cm)	75.7 (67.0-87.5)	75.7 (67.5-83.0)	0.936^2^
Hip circumference (cm)	93.46±12.88	92.6±13.08	0.843^1^
Subscapular skinfold (mm)	15.00 (10.50-16.00)	15.00 (9.7517.00)	0.567^2^
Triceps skinfold (mm)	17.00 (11.00-24.75)	20.50 (11.50-24.00)	0.643^2^
**Bioelectrical impedance (% fat mass)**			
Female	34.28±4.88	33.27±6.81	0.738^1^
Male	21.00±7.73	22.22±7.04	0.731^1^

^1^ Mean ± standard deviation, Student’s *t*-test. ^2^Median(25th and 75th percentile). Mann-Whitney test.

Table 3 shows that the biochemical parameters, including serum zonulin, were similar between the two groups of adolescents with and without fructose malabsorption. The exception was urea, which showed a statistically significant difference (*P*=0.012). When serum zonulin levels were compared between overweight and non-overweight adolescents, values of 51.75 ng/mL (14.57-52.41) and 51.89 ng/mL (49.92-52.79) were observed, respectively, and there was no statistically significant difference (*P*=0.386).

**TABLE 3:** Biochemical data and serum zonulin levels in adolescents according to the presence of fructose malabsorption.

	Fructose malabsorption		*P*
	Yes (16)	No (21)	
Fasting blood glucose (mg/dL)	86.5±12.7	84.7±6.2	0.613^1^
Fasting insulin (mg/dL)	12.2 (8.9-17.1)	9.9 (6.9-14.9)	0.610^2^
HOMA IR^3^	2.5 (1.8-3.7)	2.0 (1.4-3.3)	0.407^2^
HOMA IR >2.5	6.0 (37.5%)	5.0 (23.8%)	0.475^4^
Total cholesterol (mg/dL)	164.3±19.4	157.1±27.6	0.417^1^
LDL (mg/dL)	95.8±19.4	90.4±21.1	0.496^1^
HDL (mg/dL)	49.0±11.4	49.7±12.9	0.869^1^
Triglycerides (mg/dL)	98.6±39.6	89.8±40.4	0.541^1^
Alkaline phosphatase U/L	119.0 (84.3-162.8)	239.0 (108.5-338.5)	0.114^2^
CRP (mg/dL)	0.6 (0.0-0,6)	0.6 (0.6-1.4)	0.256^2^
VHS (mm)	4.0 (3.0-9.3)	4.0 (4.0-7.0)	0.951^2^
Urea (mg/dL)	31.7±6.3	24.5±7.8	0.012^1^
Uric acid (mg/dL)	5.1±0.8	4.6±1.1	0.195^2^
Hemoglobin (g/dL)	15.0±1.4	14.1±1.2	0.066^2^
Ferritin (ng/mL)	83.5±62.0	70.4±46.3	0.475^2^
Albumin (g/dL)	4.9±0.2	4.8±0.3	0.506^1^
Zonulin (ng/mL)	49.7(29.6-52.3)	52.2(51.0-53.3)	0.116^2^

^1^ Student *t*-test. ^2^Median (25th and 75th percentile); Mann-Whitney test. ^3^HOMA -Evaluation of the insulin resistance homeostasis model. ^4^Fisher’s exact test.

## DISCUSSION

It is important to note that this study revealed a high frequency of intestinal fructose malabsorption in asymptomatic adolescents. Fructose overload during the breath test triggered symptoms compatible with fructose intolerance in half of the adolescents with intestinal fructose malabsorption, as well as in 23.8% of those without malabsorption.

Against this backdrop, it is important to note that interest in fructose malabsorption has increased in recent decades, primarily to explain gastrointestinal symptoms associated with the condition, either occurring independently or as a part of a group of FODMAPs. In addition to these aspects, fructose may play a role in the pathophysiology of certain functional gastrointestinal disorders such as irritable bowel syndrome and functional abdominal pain in children and adolescents.

Fructose is transported through the intestinal epithelium by glucose transporter GLUT-5 and reaches the enterocyte, where part of it is catabolized by the enzyme ketohexokinase. However, most of the fructose is transferred through the basal membrane by GLUT-2 into the blood circulation of the portal system^11^. In clinical care and research, the intestinal absorption of fructose is assessed indirectly using the breath test^1^ ^,^ ^5^ ^,^ ^7^ ^,^ ^8^ ^,^ ^10^ ^,^ ^32^. The respiratory test depends on the fermentation of unabsorbed fructose by the intestinal microbiota, which produces gases- mainly hydrogen- that diffuse into the bloodstream, pass through the lungs, and are subsequently eliminated in the exhaled air. The hydrogen concentration is measured in breath samples. It is worth noting that humans are unable to synthesize hydrogen, which is produced exclusively by their microbiota^1^ ^,^ ^5^ ^,^ ^7^ ^,^ ^10^. Presumably, the bacteria in the colon are mainly responsible for this fermentation, which can be accompanied by clinical manifestations such as abdominal distension, abdominal pain, flatulence, and diarrhea, among others^1^ ^,^ ^7^ ^,^ ^9^.

There are methodological variations of how the hydrogen breath test is conducted in different research centers, although certain recommendations have been suggested^6^ ^-^ ^8^. The literature reflects discrepancies in terms of dose, number, and interval between breath sample collection, as well as the cut-off points used to define increased hydrogen concentration and characterize intestinal malabsorption^1^ ^,^ ^5^ ^,^ ^7^ ^,^ ^10^.

In the pediatric age group, most studies on intestinal fructose malabsorption have included patients with digestive diseases, particularly those with functional abdominal pain, i.e. related to gut-brain interaction disorders. Thus, our study is one of the few to evaluate fructose absorption and intolerance in adolescents with no history of gastrointestinal clinical manifestations. The frequency of fructose malabsorption in our study was high and similar to that found in three other studies^5^ ^,^ ^33^ ^,^ ^34^. For example, fructose malabsorption occurred in 50% of 34 asymptomatic Spanish children with an average age of 9 years. In this Spanish study, 71 children with functional abdominal pain were evaluated, and fructose malabsorption was recognized in 48% of them, indicating a prevalence of malabsorption similar to that of asymptomatic individuals. Also, a low-fructose diet was recommended for patients with abdominal pain and fructose malabsorption, resulting in clinical improvement in around 70% of cases^34^.

Another study evaluated 20 asymptomatic American adults and found that after ingesting a 10% solution containing 50 g of fructose, malabsorption (defined as an elevation of 20 ppm or 5 ppm in 3 consecutive samples) was observed in 16 individuals (80%), and during the test, gastrointestinal symptoms occurred in 11 individuals^5^. In the same study, the same individuals repeated the breathing test with lower doses of fructose and showed lower rates of malabsorption and fructose intolerance. Another study, also with healthy adults, showed that 58% to 87% of asymptomatic individuals showed malabsorption in the respiratory test when they ingested a 50 g dose of fructose; 10% to 53% tested positive with a 25 g dose, and 0% to 10% with a 15 g dose^33^.

A study carried out in our department showed that children with functional abdominal pain and intestinal fructose malabsorption had higher hydrogen production during the lactulose breath test, suggesting that the fermentation capacity of the intestinal microbiota of patients with fructose malabsorption is involved in fructose fermentation^10^. This difference in fermentative capacity may be directly related to the characteristics of the intestinal microbiota, as demonstrated in American children with irritable bowel syndrome^9^. It was also observed that fermentation associated with intestinal fructose malabsorption begins within the first hour, suggesting either involvement of the small intestinal microbiota in fermentation or shortened intestinal transit time. However, this aspect should be clarified in future studies with plans to simultaneously measure oral-cecal transit time with scintigraphy or another method. 

To our knowledge, there are no published studies related to the results of respiratory tests with fructose and lactulose. Thus, our results showed that adolescents without a history of gastrointestinal symptoms with fructose malabsorption showed greater hydrogen production. This was observed when they were given 10 g of lactulose for the respiratory test. This confirms the results of a study carried out in our department on children with functional abdominal pain^10^. It was also possible to observe a positive correlation between the area under the curve of the respiratory tests with fructose and lactulose (r = +0.48; *P*=0.003).

Another interesting aspect involves the gastrointestinal symptoms triggered by the ingestion of lactulose in the respiratory test. The Kappa coefficient of 0.65 showed substantial agreement between the occurrence of gastrointestinal symptoms after the administration of fructose and lactulose for the respiratory tests, even at different doses. In this context, 10 g of lactulose, which is not absorbed, appears to have the similar effect in triggering the symptoms as observed in the fructose breathing test, in which an average dose of approximately 49 g was administered, assuming partial absorption. As already mentioned, there seems to be an individual predisposition to gastrointestinal symptoms in individuals with fructose malabsorption. This may be related to variations in fermentation capacity and osmotic force, bringing liquids into the intestinal lumen, as well as visceral hypersensitivity that may facilitate the perception of symptoms related to the presence of carbohydrates in the intestinal lumen, as is observed in functional gastrointestinal diseases^1^ ^,^ ^5^ ^,^ ^7^ ^,^ ^10^. These aspects should be explored specifically in future projects.

Another important point in this study was the choice of the habitual dietary survey, which revealed that fructose intake was similar for adolescents with intestinal fructose malabsorption as for adolescents without this condition. The average daily fructose consumption by adolescents was approximately seven times lower than the dose administered in the breath test. This may explain the lack of a history of gastrointestinal symptoms among adolescents. In addition, the results of our study confirmed the possibility of gastrointestinal symptoms being triggered after fructose ingestion in 10 to 20% of healthy individuals with no fructose malabsorption as evidenced by the respiratory test^5^; however, abdominal pain was more frequent in adolescents with fructose malabsorption (*P*=0.023). Notably, poor fructose absorption in the breath test was not associated with lower habitual fructose intake.

In the present study, there was no association between fructose malabsorption and increased body adiposity as measured by anthropometric parameters and bioelectrical impedance, which contradicts the results found in a previous study carried out in our department, which showed higher body mass index (BMI) values in children with abdominal pain and fructose malabsorption^10^. However, it is important to point out that in this previous study, the average fructose intake of patients with fructose malabsorption was higher than that of patients without fructose malabsorption, which is surprising, as well as being a possible alternative explanation for the increase in BMI. In turn, 9 obese pediatric patients with non-alcoholic fatty disease and 6 patients with obesity showed greater fructose absorption, i.e. lower hydrogen production in the respiratory test, when compared to 9 children who were not overweight^35^. From a metabolic point of view, the administration of fructose was associated with higher levels of glycemia, insulinemia, and uric acid, leading to the conclusion that obese children with liver disease absorb and metabolize fructose more effectively.

In this context, a positive correlation was observed in American adults of African descent, between intestinal fructose absorption and fat content in the liver^36^, suggesting that individual fructose absorption capacity may be related to metabolic profile and body fat.

In our study, no difference was observed in the metabolic parameters of adolescents with and without intestinal fructose malabsorption, except for blood urea levels, which were higher in adolescents with fructose malabsorption. However, no plausible explanation for this finding has been found in the literature. Furthermore, changes in fructose consumption and GLUT-5 expression in the intestine may contribute to the development of intestinal and metabolic diseases through alterations in the intestinal microbiota or intestinal barrier^37^. Increased blood levels of zonulin have been reported in obese children^19^ ^,^ ^20^ and adolescents^21^, which may be associated with increased intestinal permeability. However, in the present study, serum zonulin levels did not differ significantly between overweight and non-overweight adolescents.

Our study, like others on the topic, has some limitations. A convenience sample was evaluated, based on the demand for care at the service during the data collection period. This requires caution when extrapolating the results to other groups of adolescents. On the other hand, the sample was sufficient to show significant differences in the adolescents’ responses to the lactulose administration according to the occurrence of fructose malabsorption.

In conclusion, according to the breath test, a high frequency of fructose malabsorption was observed in adolescents with no history of gastrointestinal clinical manifestations. Half of the adolescents with fructose malabsorption and approximately one-quarter of the adolescents without malabsorption showed symptoms compatible with the condition. Fructose malabsorption did not influence the estimate of habitual fructose consumption. Although a diet rich in fructose and the expression of GLUT-5 influence various types of systemic diseases^37^. Based on the biochemical parameter study, the present study did not identify an association between fructose malabsorption and body adiposity, intestinal permeability, or metabolic alterations.

## Data Availability

Data-available-upon-request
